# Novel insight into pancreatic adenocarcinoma pathogenesis using liquid association analysis

**DOI:** 10.1186/s12920-022-01174-3

**Published:** 2022-02-18

**Authors:** Zahra Shokati Eshkiki, Nasibeh Khayer, Atefeh Talebi, Reza Karbalaei, Abolfazl Akbari

**Affiliations:** 1 Alimentary Tract Research Center, Clinical Sciences Research Institute, Ahvaz Jundishapur University of Medical Sciences, Ahvaz, Iran; 2 Skull Base Research Center, The Five Senses Health Institute, Iran University of Medical Sciences, Tehran, Iran; 3 Colorectal Research Center, Iran University of Medical Sciences, Tehran, Iran; 4 Department of Psychology and Neuroscience Program, Temple University, Philadelphia, PA USA

**Keywords:** Pancreatic ductal adenocarcinoma, Liquid association analysis, Three-way gene interaction, Gene set enrichment analysis, Therapeutic targets

## Abstract

**Background:**

Pancreatic ductal adenocarcinoma (PDAC) is a lethal malignancy associated with a poor prognosis. High-throughput disease-related-gene expression data provide valuable information on gene interaction, which consequently lead to deeper insight about pathogenesis. The co-expression analysis is a common approach that is used to investigate gene interaction. However, such an approach solely is inadequate to reveal the complexity of the gene interaction. The three-way interaction model is known as a novel approach applied to decode the complex relationship between genes.

**Methods:**

In the current study, the liquid association method was used to capture the statistically significant triplets involved in the PDAC pathogenesis. Subsequently, gene set enrichment and gene regulatory network analyses were performed to trace the biological relevance of the statistically significant triplets.

**Results:**

The results of the current study suggest that “response to estradiol” and “Regulation of T-cell proliferation” are two critical biological processes that may be associated with the PDAC pathogenesis. Additionally, we introduced six switch genes, namely *Lamc2*, *Klk1*, *Nqo1*, *Aox1*, *Tspan1,* and *Cxcl12*, which might be involved in PDAC triggering.

**Conclusion:**

In the current study, for the first time, the critical genes and pathways involved in the PDAC pathogenesis were investigated using the three-way interaction approach. As a result, two critical biological processes, as well as six potential biomarkers, were suggested that might be involved in the PDAC triggering. Surprisingly, strong evidence for the biological relevance of our results can be found in the literature.

**Supplementary Information:**

The online version contains supplementary material available at 10.1186/s12920-022-01174-3.

## Introduction

It is well established that adenocarcinoma (PDAC) is responsible for more than 90% of all pancreatic cancer cases [1]. The median survival duration of PDAC is less than 6 months, and only 6% of patients survive by passing 5 years from their diagnosis. Unfortunately, due to late diagnosis and resistance to the currently available chemotherapy drugs, the proposed treatments have not improved significantly [2, 3]. Therefore, obtaining a better understanding on molecular mechanisms of the disease’s progression and discovery of novel therapeutic targets is an extremity needed to improve the treatments of PDAC. Although the pathogenesis of PDAC is extensively studied, it remains unknown yet.

Gene interactions provide critical information on disease pathogenesis. In this regard, high-throughput gene expression data is widely adopted for elucidating gene interactions [4, 5]. Accordingly, such data have been extensively investigated to explain the pathogenesis of PDAC [6]. Giulietti et al. have suggested several central genes involved in PDAC using the weighted gene co-expression network analysis on the PDAC microarray-based gene expression data. Additionally, they proposed several biological processes, including lipid metabolism and transmembrane transport, which are critical in the PDAC pathogenesis [7]. On the other hand, Zhou et al. have introduced several hub genes associated with both the progression and prognosis of PDAC by constructing a co-expression network. Besides, they have suggested that the cell cycle plays a vital role in PDAC [8].

To the best of our knowledge, a large amount studies to detect essential genes and pathways involved in PDAC were performed using the two-way interaction approach [7–9]. Such an approach can capture co-expressed genes, may possibly encode some functionally-related proteins. However, sometimes the two-way interaction approach is inefficient to trace functionally-related genes. A reason for this fact is the molecular state of a cell is susceptible to intra- and inter-cellular alteration. Therefore, the co-expression relationship of two functionally-related genes may change based on the cellular condition. In other words, the co-expression relationships may have a dynamic nature depending on the cellular state. Hence, the two-way interaction approach is too simplistic to describe the complex relationships among genes [10–12].

The three-way interaction approach is a more efficient strategy to trace functionally-related genes. Such an approach is inferred from the cross-shaped co-expression pattern, which in the expression levels of two genes is directly correlated under a certain condition, while these are inversely correlated under another condition. More precisely, the three-way interaction approach captures the dynamic nature of the co-expression pattern using introducing the third gene, called a switch gene. In other words, depending on the expression level (or genotype) of the switch gene, the expression levels of a gene pair are either directly or inversely correlated [13–15]. Therefore, determining the three-way interaction would be considered a pivotal step to decode the complex biological systems.

Moreover, the three-way interaction approach can be a competent compromise between the reality of the complexity of biological interactions and the discoverability of interactions. Because the two-way interaction approach is too simplistic for explaining complex molecular interactions; on the other hand, any four-way or higher-level models are less utilizable because, in the transcriptomics level, these models require a huge computational demand (as the number of combinations increases exponentially with the number of interaction levels) [13, 16].

A well-known representative of the three-way interaction is the relationship among thyroid hormone expression, growth hormone, and thyroid hormone receptor (TR-RXR). TR-RXR plays an essential role as a suppressor or as an activator depending on the absence or the presence (amount) of thyroid hormone, respectively. Therefore, it can be stated that the expression levels of both growth hormone and TR-RXR are directly correlated in the presence of thyroid hormone, but they are inversely correlated in the absence or with the low amount of thyroid hormone, as a switch gene [17].

**Fig. 1:**
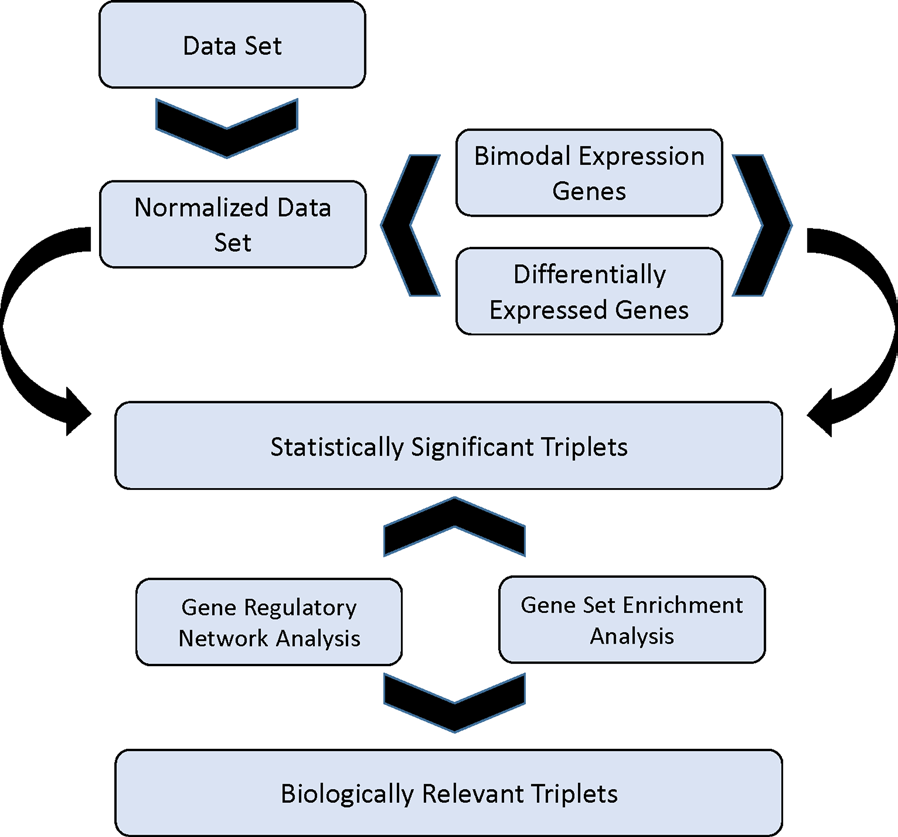
Flowchart for identification biologically relevant three-way interaction

To the best of our knowledge, gene expression relationships in PDAC have not been studied using the three-way interaction model so far. Therefore, the present study aimed to investigate three-way interaction in the PDAC-related microarray gene expression data, in order to find the potential biomarkers and biological processes involved in their pathogenesis (Fig. 1).

## Materials and methods

### Choosing the dataset and reducing the dimension

In order to gather PDAC associated gene expression profiling datasets, the publicly available transcriptome data repositories include ArrayExpress [18], NCBI gene Expression Omnibus (GEO) [19] and The Cancer Genome Atlas (TCGA) [20], were comprehensively screened. Among numerous transcriptome datasets such as GDS4336 [21] and GSE15471 [22], carried out with PDAC and adjacent non-tumor tissue samples, we preferred to employ GDS4336 considering its large sample size and sampling characteristic.

The employed databases included pancreatic ductal adenocarcinoma tumour (45 samples) and adjacent non-tumour tissue gene expression (45 samples). There were 35,556 probe sets on Affymatrix Human Gene 1.0 ST Array [23]. Thereafter, we used the genefilter package [24] in Bioconductor to remove the duplicate probes. Finally, the 18,232 genes remained for further statistical analyses.

### Selecting the candidate switching genes

According concept the three-way interaction, a {X_1_, X_2_} gene pair have a contradictory co-expression behavior depending on the expression level of corresponding switch gene. Therefore, it is expected a disease related-switch gene exhibit the following traits (1) differentially expressed in a disease samples compare to healthy ones and consequently, (2) its gene expression distribution be bimodal.

Thereafter, fold change and standard errors were estimated by fitting a linear model (using the lmFit function in Limma package [25] for each gene in the groups. Genes with empirical Bayes t-test and *p* values of 0.05 were firstly selected and then corrected by calculating the false discovery rate *p* < 0.05. Furthermore, the bimodality of each one of the gene’s expression was calculated by diptest function in diptest package [26] in the selected genes by considering *p* < 0.05.

In this way, the two above-mentioned constraints were applied to define the candidate switching genes.

### Determining the liquid association triplets

The three-way interactions between the candidate switching genes as well as all the possible combinations were calculated using the fastMLA function in the fastLiquidAssociation package [27]. Accordingly, this package works using a modified liquid association algorithm for the determination of changes in the co-expression relationships of the other two genes X_1_ and X_2_, in terms of the expression level of the third gene (X_3_). Notably, before running the liquid association algorithm, performing two pre-processing stages for each variable on the data are essential, which are as follows: the first is transforming into normal distribution [28], and the second one is standardizing the mean to 0 and standard deviation to 1 [29]. In the present study, the first and second steps were performed by an in-house script and CTT package [30], respectively. Next, the Bonferroni correction approach was used to estimate the false discovery rate (FDR) [31]. As well, the statistically significant triplets were considered the three-way interactions with FDR < 0.05.

### Constructing the gene regulatory network

One of the fundamental properties of the gene regulatory network (GRN) is providing a systematic proficiency regarding the complicated molecular mechanisms, which can be used to check gene expression under diverse biological conditions [32, 33]. The geWorkbench_2.6.0 software was then applied to reconstruct GRN among all the studied genes in the dataset as hub markers. Moreover, the Algorithm for the Reconstruction of Accurate Cellular Networks (ARACNE) was used to construct the GRN [32]. Accordingly, this algorithm is able to make mutual information (MI) as a measure to discover the direct regulatory interactions between each transcriptional regulator and its potential target(s).

### Gene set enrichment analysis

Gene set enrichment analysis (GSEA), which is a popular statistical method, can establish the validity of the shared association among a set of genes using a predefined Gene Ontology [34]. Afterward, GSEA was done in this study using the Biological Process of Gene ontology database [35]. These analyses were applied using the ClueGO plugin [35] within the Cytoscape v.3.3.0 environment [36]. Additionally, the two-sided hypergeometric test along with the Bonferroni step-down correction approach and a kappa score level of 0.4 were regarded as the statistically significant analyses.

### In silico validation in other related and unrelated gene expression datasets

The SurvExpress [37] web-based biomarker validation tool was used to assess the suggested switch genes as potential biomarkers in various cancer types. Validation analyses were carried out through related as well as unrelated gene expression datasets obtained from TCGA [20], ICGC [38] and NCBI-GEO [19].

The suggested switch genes; namely, *Lamc2*, *Klk1*, *Nqo1*, *Aox1*, *Tspan1,* and *Cxcl12*, were surveyed through pancreatic ductal adenocarcinoma (ICGC, n = 189), cervical squamous cell carcinoma (TCGA, n = 191), lymphoma (NCBI-GEO, GSE10846, n = 420), glioblastoma (TCGA, n = 148), hepatocellular carcinoma (NCBI-GEO, GSE10143, n = 162) and stomach adenocarcinoma (TCGA, n = 352).

For each utilized dataset, samples according to their prognostic index were classified into low and high-risk groups. Subsequently, in each dataset, the prognostic performance of suggested switch genes was determined using Kaplan–Meier plots, log-rank test *p* values, hazard ratios (HR), and their confidence intervals (CI). Additionally, the correlation of the survival analysis with gene expression levels was presented by Heatmaps, which in samples were sorted by their prognostic index, and genes were clustered based on Euclidean distance. Moreover, differences in gene expression levels between high and low-risk groups were determined using the t-test; furthermore, box plots were created to illustrate such differences.

### Ethical approval statements

Since the current investigation that was performed using a public GEO dataset (accession number: GDS4336) can be considered a computational survey, obtaining both ethics approval and consent statements was not required in this research.

## Results

### Data pre-processing and candidate switch gene selection

After pre-processing and normalization of the obtained data using the robust multi-array analysis method, the final dataset consisted of 90 samples, including 45 pancreatic ductal adenocarcinoma tumour samples and 45 adjacent non-tumour samples. Accordingly, each Array included 28,896 probes. After removing duplicate probes, this number dropped to 18,232 probes.

After restricting the dataset to include both the differentially expressed genes and bimodality expressed genes, the 53 genes were remained. Such genes considered as the candidate switch genes to be analysed using fastLA. The list of the candidate switch genes is reported in the Additional file 1: Table S1.

### Tracing statistically significant cross-shaped triplets

**Fig. 2:**
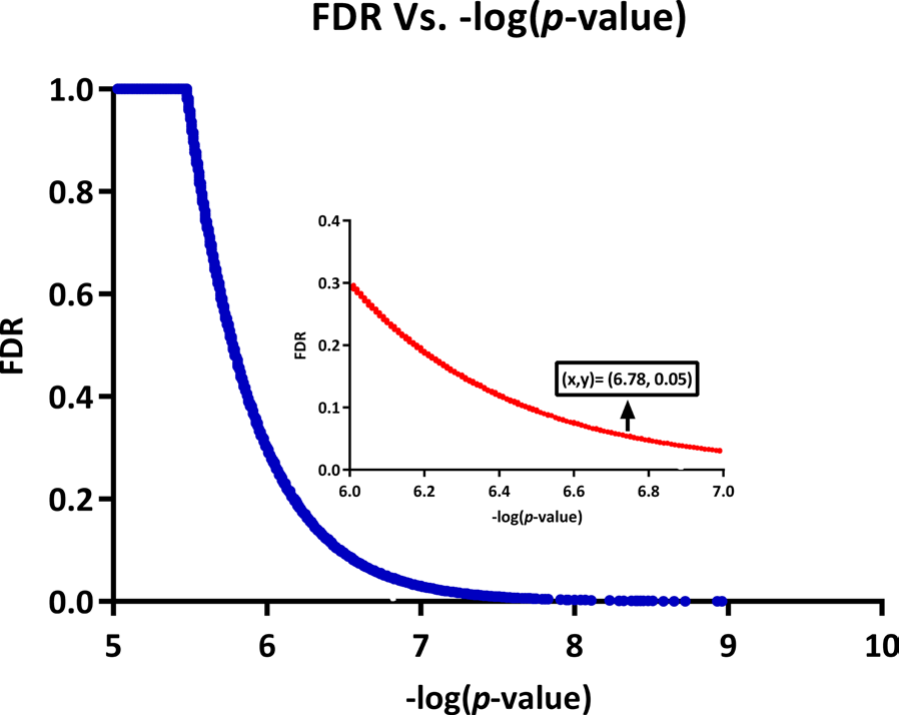
FDR versus − log (*p*-value). The changes in FDR (Bonferroni-corrected *p*-value) versus − log (*p*-value) for the first 10,000 results of fastLA [21]. As shown FDR = 0.05 corresponds to − log (*p*-value) = 6.78

The liquid association analysis was performed for each combination consisting of a candidate switch gene (X_3_) and each possible pair of genes in the dataset {X_1_, X_2_}. Subsequently, we selected the top 300,000 triplets with the highest significance levels in terms of their *p*-values, which were defined as the outputs of this analysis. Figure 2 shows the changes in FDR versus − log (*p*-value) for the first 300,000 triplets. In addition, a set of significant cross-shaped triplets comprising of 220 triplet combinations were chosen for conducting more analyses, in terms of FDR < 0.05. The list of the statistically significant triplets is reported in the Additional file 1: Table S2.

### Identifying the biological process involved in PDAC

**Fig. 3:**
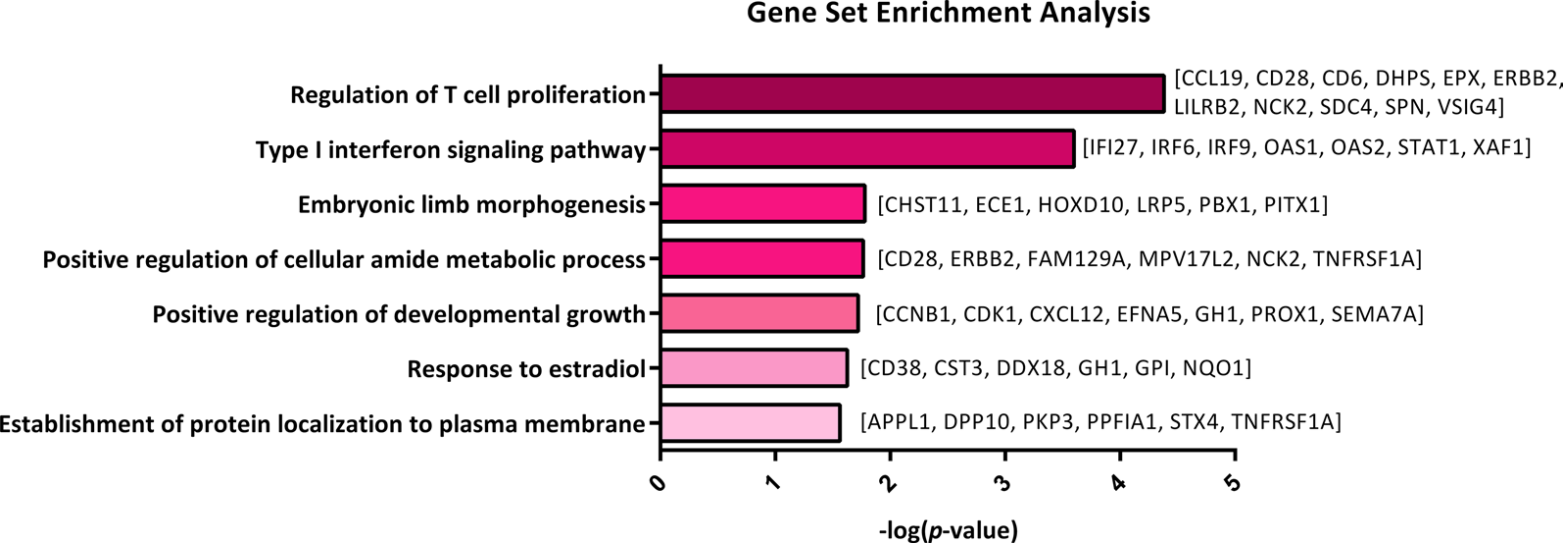
Gene set enrichment analysis (GSEA). Enriched terms based on biological process for all genes involved in statistically significant triplets. The biological relevance of two statistically significant triplets was confirmed by GSEA

GSEA was applied to detect the biologically-relevant triplets as well as finding the BP involved in PDAC. Correspondingly, GSEA was performed with a *p* value < 0.05 and an FDR < 0.1 for all the genes involved in the statistically significant triplets. Since the terms in lower levels of gene ontology are more general, so the enriched terms in levels lower than five were not reported. Figure 3 shows all the significantly enriched terms, including “Response to estradiol”, “Embryonic limb morphogenesis”, “Positive regulation of cellular amide metabolic process”, “Establishment of the protein localization to plasma membrane”, “Type I interferon signalling pathway”, “Positive regulation of the developmental growth”, and “Regulation of T cell proliferation”.

According to the definition of the three-way interaction, gene expressions of both X_1_ and X_2_ were found to be directly correlated under a specific condition. So, it is expected that both X_1_ and X_2_ be in a common pathway or biological process. Therefore, in the current study, we detected the statistically significant triplets in the enriched terms to identify the biologically relevant triplets among them. In this regard, we identified two biologically relevant triplets in terms of GSEA, including* Klk*, and {*Cst3, Gh1*}, which are involved in the "Response to estradiol" process, as well as *Lamc2* and {*Ccr7, Dhps*}, which are involved in the "Regulation of T cell proliferation" process.

**Fig. 4:**
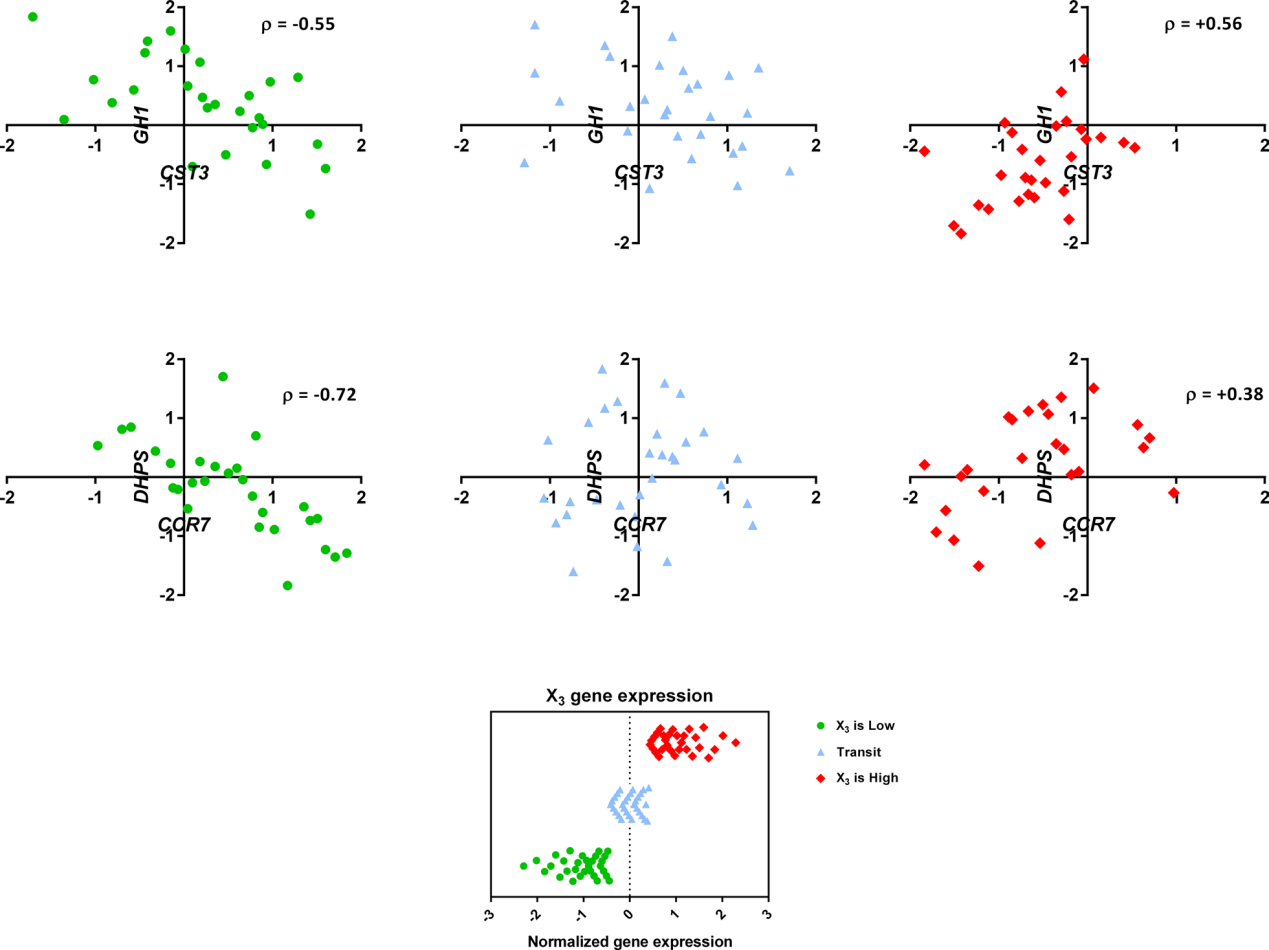
Scatter plot of two biologically relevant triplets. Based on the fastLA algorithm, the samples are divided into three-bin according to the expression of the X_3_ gene. In each case, a considerable change in the correlation of X_1_and X_2_ occurs as a result of the change in X_3_

Additionally, Fig. 4 presents the scatter plot of these triplets. This plot also reveals a remarkable change in the correlation between X_1_ and X_2_ resulting from the changes in X_3_ expression level.

### Detecting the cross-shaped triplets in GRN

In order to further analyse the functional relevance of the statistically significant triplets, a GRN was constructed using ARACNE. The detailed information of such network is reported in the Additional file 1: Table S3. According to the concept of the three-way interaction, the expression level of the switch gene (X_3_) affects the correlation direction of the {X_1_, X_2_} gene pair. In this regard, it is expected to observe a regulatory relationship between these genes.

**Fig. 5:**
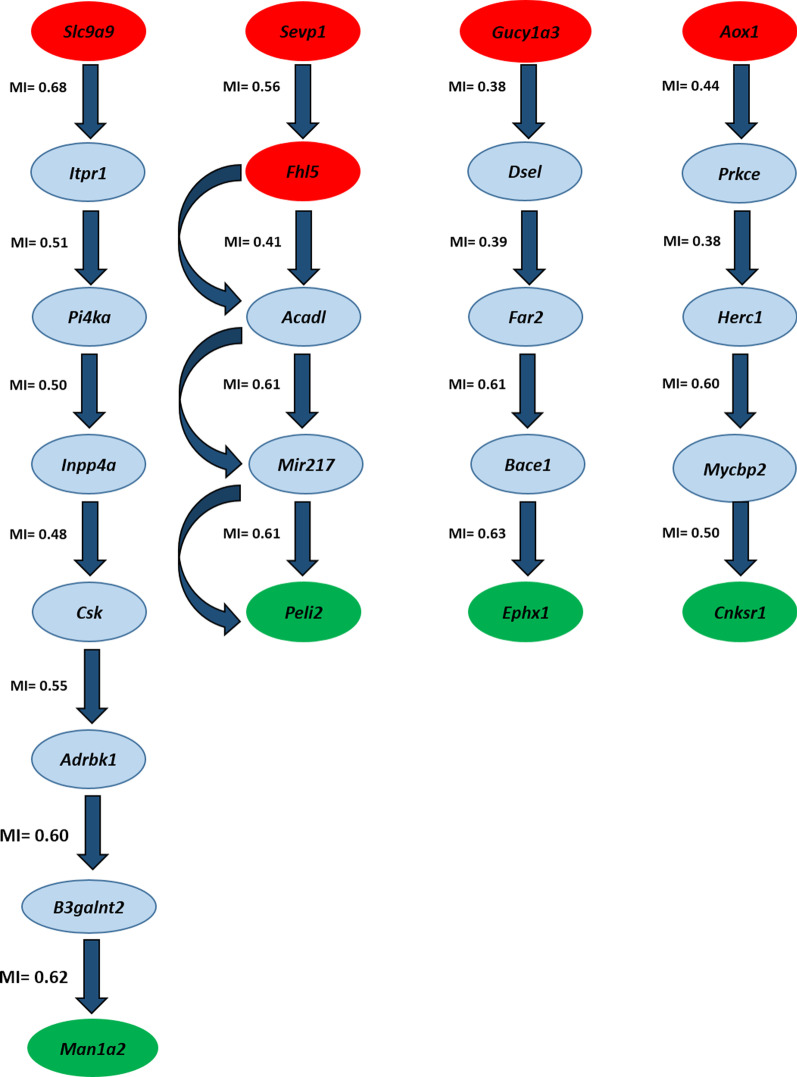
Regulatory relationships within triplets. The regulatory relationships of significant triplets obtained from liquid association analysis were traced in the GRN. The Mutual information (MI) value identifies the magnitude of each relationship

By detecting the statistically significant triplets in GRN, we found that seven triplets may be biologically relevant to each other. The position of such triplets is presented as a sub-network of GRN in Fig. 5. The information on the liquid association analysis of such triplets is reported in Table 1.

**Table 1:** The liquid association analysis information of the seven triplets that the regulatory relationships of them were traced in gene regulatory network

X_1_ or X_2_	X_2_ or X_1_	X_3_	Rhodiff	MLA value	Wald	*p* value	Bonferroni
PELI2	FHL5	NQO1	1.0672	0.3849	30.5523	3.25E−08	9.75E−03
CNKSR1	NSUN6	AOX1	1.1351	0.4016	29.7896	4.82E−08	1.45E−02
PELI2	FHL5	AOX1	− 1.2243	− 0.4384	28.9932	7.26E−08	2.18E−02
PELI2	FHL5	TSPAN1	1.178	0.413	28.4857	9.44E−08	2.83E−02
EPHX1	GUCY1A3	TSPAN1	1.1812	0.4154	28.3721	1.00E−07	3.00E−02
PELI2	SVEP1	AOX1	− 1.2403	− 0.44	27.9954	1.22E−07	3.66E−02
MAN1A2	SLC9A9	CXCL12	− 1.1935	− 0.4272	27.5945	1.50E−07	4.50E−02

Altogether, the biological relevance of nine triplets was approved by either GSEA or GRN methods.

### The specificity of the switch genes to PDAC

In order to in-silico validation of suggested switch genes as PDAC's potential biomarker, several analyses were carried over various human cancers.

**Fig. 6:**
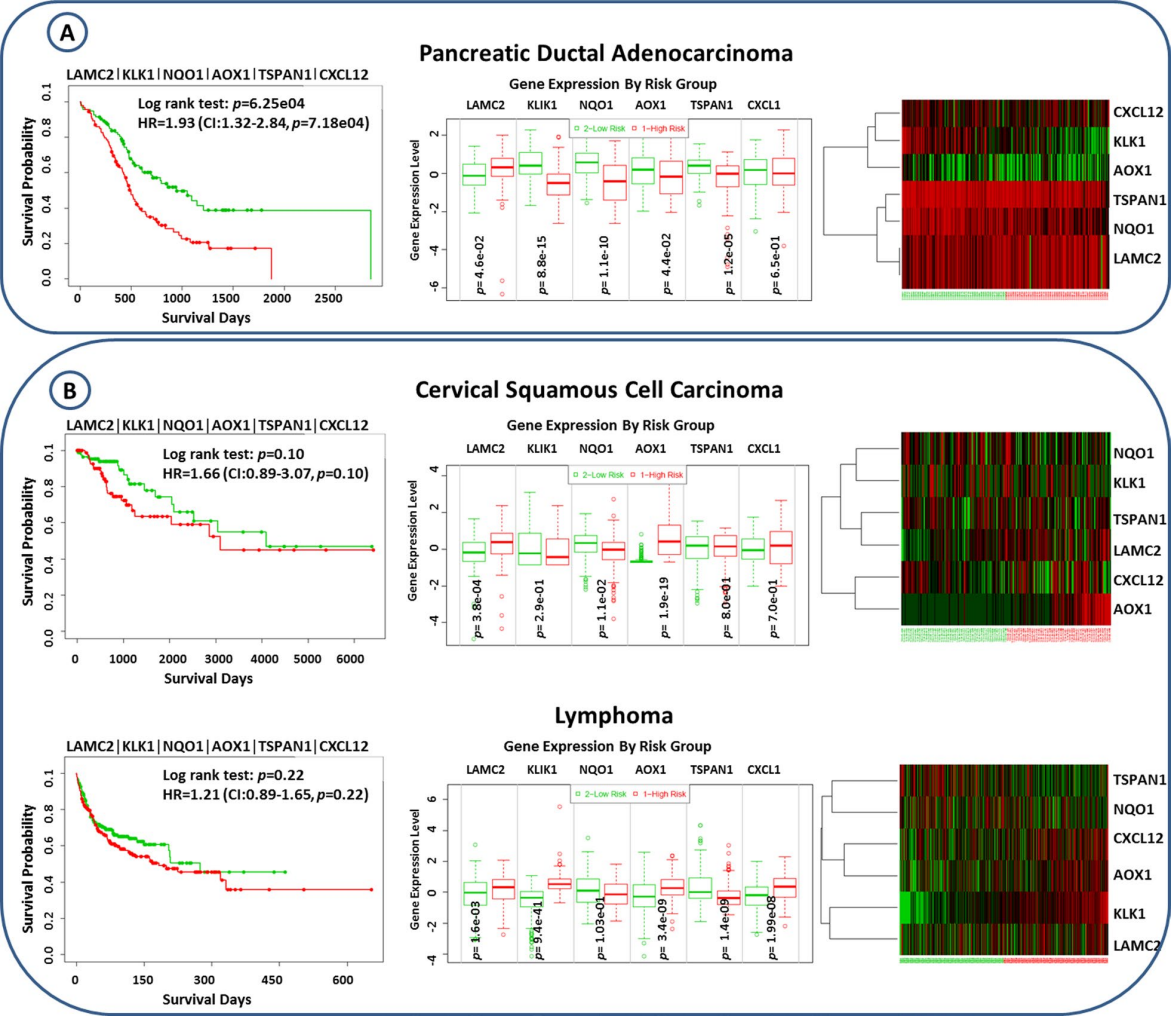
The prognostic power of suggested switch genes through related and unrelated datasets. (**A**) Pancreatic ductal adenocarcinoma as a related dataset, (**B**) cervical squamous cell carcinoma and lymphoma as two exemplary unrelated datasets

First of all, the prognostic performance of suggested switch genes was assessed in a related gene expression dataset (i.e., PDAC). As indicated in Fig. 6a, the suggested switch genes show significant prognostic performance in such a PDAC-related dataset (HR = 1.93, *p* = 7.8 × 10^−5^). Then, the prognostic performance of suggested switch genes was assessed in five gene expression datasets of unrelated but highly prevalent cancers. The survival analyses pointed out insignificant performance in such cancers, cervical squamous cell carcinoma (*p* = 0.10), lymphoma (*p* = 0.22), glioblastoma (*p* = 0.06), hepatocellular carcinoma (*p* = 0.87) and stomach adenocarcinoma (*p* = 0.10). (Additional file 1: Fig. S4). As a consequence, our suggested switch genes could be considered as prognostic for PDAC.

The prognostic power of suggested switch genes in cervical squamous cell carcinoma and lymphoma as two exemplary datasets shown in Fig. 6b. The survival analysis results of other PDAC-unrelated datasets were presented in Additional file 1: Fig. S4.

## Discussion

The improvements are seen in our descriptive comprehension of pancreatic ductal adenocarcinoma; however, some considerable cancer biology features like the entire involved genes, still remain poorly understood. It is obvious that obtaining a better understanding on PDAC biology may lead to provide more effective treatments in this regard [39]. Herein, we used a novel bio-computational approach to identify new candidate genes involved in this aggressive cancer process. To the best of our knowledge, the present study introduced a novel bio-computational approach for the first time, named as the three-way interaction model, to investigate new candidate genes as well as the possible mechanisms of the gene switching in PDAC.

### Gene set enrichment analysis

The enriched biological processes (BP) are “response to estradiol”, “embryonic limb morphogenesis pathway”, “positive regulation of cellular amide metabolic process”, “establishment of protein localization to plasma membrane”, “type I interferon signalling pathway”, “positive regulation of the developmental growth”, and “regulation of T-cell proliferation”. As well, all the seven common enriched terms are biologically relevant to PDAC. For more information, see below.

In this regard, the first enriched biological process is the "response to estradiol". Pancreatic cancer cell proliferation is highly estrogen-sensitive in vitro, and estrogen receptor alpha and beta are frequently expressed in cancerous cells [40]. ERbeta/ERalpha ratio may even affect the estrogen-mediated growth stimulation and then reduce cytotoxicity at physiological concentrations that may possess some clinical implications for pancreatic cancer therapy [40]. A recent study demonstrated the critical roles of estrogen receptors and the efficacies of anti-estrogen therapy in both pancreatic cancer cell’s proliferation as well as cancer progression’s inhibition [41]. In some previous studies, it was suggested that T-box factors, especially Tbx2, as transcription factors, play important roles in controlling cell cycle progression, embryonic development, and cancer genesis. Tbx2 is implicated in various developmental processes like the morphogenesis of various organs and tissues such as limbs, kidneys, lungs, heart. Moreover, it was shown that it is overexpressed in various types of cancers such as pancreatic, liver, bladder, and breast cancers and can overwhelm senescence, which is a cellular process serving as a cancer development inhibitor [42].

Another enriched biological procedure is "positive regulation of cellular amide metabolic process". Nitrogen-based nucleotides synthesis and nonessential amino acids (NEAAs) are known as the essential metabolic stages in tumour cell growth. Accordingly, by donating its amide group and converting it into glutamic acid, it can provide an essential nitrogen donor in 3 autonomous enzymatic reactions in charge of purine synthesis. In addition, it is responsible for pyrimidine synthesis in two genes [43, 44]. Hence, the amide metabolism pathway and the change of glutamine from an NEAA in normal cells into an essential amino acid within cancerous cells play the roles of the main substrates feeding the tumorigenic cells’ anabolic growth procedures.

Interestingly, a previous study [44] indicated that in PDAC cells, both amide and glutamine metabolism pathways support tumorigenic growth through the noncanonical metabolic pathway, which is oriented by the general biochemical position adopted by several types of cancer cells [45].

"Establishment of the protein localization to plasma membrane" is another enriched biological process. Such a biological process is considered an underlying mechanism for increasing the aggressive performance of pancreatic cancer. Accordingly, it was identified that some localized proteins, like the protein placenta-specific 8 (PLAC8, Onzin), are related to the pancreatic tumour progression. Furthermore, these can be expressed invasively and ectopically within the human PDAC and advanced preneoplastic lesions. Nevertheless, the precise molecular function of the localized proteins is still unclear, and some pieces of evidence highly proposed its role, which greatly is based on both physiologic and cellular contexts. It was demonstrated that such proteins are localized to the plasma membrane in pancreatic cancer cells, within which they interact with particular membranous structures in a spatially and temporally stable mode [46].

As mentioned earlier, "Type I interferon signalling pathway" is another enriched biological process. It was shown that Type I interferons (e.g., IFNα/β) possess various antitumor activities. Some clinical studies evaluated the effects of adjuvant IFNα treatment on the obtained equivocal findings regarding pancreatic cancer [47]. Importantly, unlike permissive PDAC cell lines, the resistant PDA cell lines can mainly respond and secrete type I interferon (IFN). Correspondingly, this indicates the contribution of the intact type I IFN responses to their resistance phenotype [48]. However, it is thought that cancer cells are mostly defective in type 1 IFN responses and also in production [49, 50]. As well, since IFN responses are normally pro-apoptotic, anti-proliferative, and anti-angiogenic [51], they are undesirable circumstances for tumour formation. Nevertheless, some cancer cells, such as some mesotheliomas [52], melanomas [53], lymphomas [54], bladder cancers [55], renal cancers [56], and possibly other types of cancers [47, 57], create or respond to type I IFN [58]. Therefore, despite the fact that human pancreatic cancer cell lines variably respond to IFNα and β, it seems that both type I interferon signalling pathway and the expression level of the type I IFN receptor play an important role in pancreatic cancer progression.

The "positive regulation of developmental growth" is also another enriched biological process. Scientifically, the idea of cancer cells sharing some features of their embryonic predecessors is historical [59]. Tumour cells, or at least a subset of them, similar to embryonic development, can preserve indefinite growth and cellular plasticity. It was indicated that the developmental paths could direct the start of PDA precursors from their cellular ancestors; however, embryonic signalling paths such as TGFβ, Hedgehog, and Wnt-β-catenin alone are insufficient for the PDA initiation [60]. Recent data displayed the role of Sox9, which is expressed throughout the development, in the PDA initiation [61, 62]. Therefore, the positive regulation of the developmental growth pathway is associated with pancreatic cancer progression.

The last enriched biological process is "regulation of T-cell proliferation". It was shown that immune disorders are one of the most significant hallmarks of cancer, and pancreatic cancer is characterized by abnormal immune cell infiltration. Accordingly, this is related to the immunosuppressive circumstances facilitating the tumour cells’ escape from immune cells [63, 64]. In pancreatic cancer, tumour-infiltrating regulatory T-cells (Tregs) are considered independent prognostic factors, which increase the secretion of different immunosuppressive cytokines, prevent cytotoxic lymphocyte function, and play a role in the immune escape [65–67].

### Biologically relevant triplets

In this study, among the statistically significant triplets, two triplets whose X_1_ and X_2_ genes are involved in the same biological procedure are known as the biologically-relevant triplets.

#### The relationship between the involved genes in triplet *Klk1* and {*Gh1*, *Cst3*}

One of the biologically relevant triplets contains Kallikrein-1 (*Klk1*), as the switch gene, and a gene pair {growth hormone 1 (*Gh1*), cystatin C (Cst3)} (Fig. 4). According to Fig. 3, genes in this triplet are involved in the cancer’s progression via "response to estradiol" biological process. It was also demonstrated that estrogens, like estradiol, are a major factor in the cancer’s progression, like breast cancer [68]. Additionally, these are tightly linked to the growth hormone (Gh/Gh1) [68].

Gh1 is a pituitary peptide hormone with some stimulating pleiotropic effects. The secretion of Gh1 occurs through two systems, including autocrine and paracrine. In human beings, Gh1 signal transduction not only is primarily mediated via binding to the GH receptor (GHR), but it could also be done by the prolactin receptor (PRLR) [69]. In several types of cancers like triple-negative breast cancers (TNBC), a correlation exists between a higher expression of the membrane-bound G protein‑coupled estrogen receptor (GPER) and a worse consequence caused by that. On the other hand, a strong connection potentially exists between GPER expression and growth hormone receptor (GHR). Previously, Girgert et al. in their study have reported that the inhibition of GH receptor reduces the expression of G protein‑coupled estrogen receptor (GPER) and inhibits growth stimulation of breast cancer indeed via the inhibition of estrogen (estradiol) signal transduction [68].

The *Cst3* gene belongs to the cystatin superfamily that encompasses some proteins containing multiple cystatin-like sequences, which are considered endogenous inhibitors of lysosomal cysteine proteinases. Moreover, it was found that this inhibitor contributes to various pathological and normal processes and seems to be implicated in the malignant progression of different human tumours. The increased *Cst3* levels have also been reported in patients with malignant diseases [70, 71]; however, the exact role of *Cst3* in malignant diseases is still debatable [70]. It has been previously reported that hormones, including *Gh1* [72, 73] and steroidal agents (estradiol), can increase *Cst3* levels in the circulation [70]. Therefore, the increased expression levels of both *Gh1* and *Cst3* (as a positive co-expression) and cross talk between the factors, especially via responding to the estradiol pathway, may play a key role in the progression of cancer [73]. Therefore, as displayed in the above-mentioned pieces of evidence, a direct co-expression relationship exists between the *Gh1* and *Cst3* genes. So, those pieces of evidence confirmed our results.

The human kallikreins, as a cluster of 15 serine protease genes, are located in the chromosomal band 19q13.4. It is considered a non-randomly reset area in numerous solid tumours like pancreatic cancer [74]. Such family genes are thought to play a role in the tumorigenesis process due to their high expression profile in hormone-dependent cancers (HDCs), in their regulation by the steroid hormones like estradiol, and in their processing abilities that have been found to be associated with tumour’s progression [75]. In comparison to pancreatic juices obtained from PDAC patients with noncancerous controls, a previous study [76] recognized some differential proteins in cancer samples like kallikrein 1 (*Klk1*). Accordingly, this indeed indicates the importance of this factor in the PDAC progression [77]. Previously, Jones et al. in their study proved that the release of kinins is catalyzed by rat Klk1 that is able to stimulate both prolactin and Gh1 secretion [78]. The *Klk1* gene was also observed to be associated with prolactin-secreting cells within human Gh1-secreting adenomas [79]. Thus, as we found in the current study, in hormone-dependent cancers, *Klk1*, as the switching gene, can indirectly regulate both *Gh1* and *Cst3* and also provide a target for promotor-specific therapeutics in PDAC.

#### The relationship between the involved genes in triplet ***Lamc2*** and ***{Dhps, CCr7}***

Another biologically relevant triplet contains the laminin subunit gamma 2 (*Lamc2*) gene, as the switch gene, as well as a gene pair [deoxyhypusine synthase (*Dhps*), chemokine receptor 7 (*Ccr7*)] (Fig. 4). All the genes involved in such triplet were shown to participate in the cancer’s progression via the regulation of the "T-cell proliferation" pathway (Fig. 3). Recently, the relationship between T-cell conversion and some mutations contributing to an immunosuppressive tumour’s microenvironment within the immune escape of pancreatic cancer, has been proved [67, 80].

It was previously indicated that the binding of the chemokines to the chemokine receptor affects the signal transduction of the T lymphocytes activation pathway. This signal pathway plays a crucial role in the cancer’s progression because it will lead to an affinity increment of a surface adhesion molecule, which is known as integrins, and enhance T lymphocyte extravasation via capillary walls [81]. The C–C chemokine receptor type 7 is a protein, encoded by the *Ccr7* gene in human beings. For this receptor, two ligands were recognized, including the (C–C motif) ligand 21 (CCL21) and chemokines (C–C motif) ligand 19 (CCL19/ELC). In this regard, concerning *Ccr7*, the relationships between this chemokine receptor expression in lymph node and tumour cells metastasis were represented in various types of cancers such as colorectal [82], gastric [83], esophageal [84], hepatocellular [85], thyroid cancer [86], malignant melanoma [87], breast cancer [88], cervical cancer [89], and pancreatic cancer [90]. Moreover, it is well-known that by ligating both the chemokines *CCL21* and *CCL19* to *Ccr7*, the homeostatic homing of T-cells is regulated into secondary lymphoid organs (SLOs) [91]. Recent studies performed on cancer revealed the essential roles of *Ccl21-Ccr7* in neoangiogenesis, proliferation, activation, retention, and accumulation of T-cells at the persistent inflammation sites [92, 93].

Deoxyhypusine synthase (Dhps) through hypusine formation leads to the activation of eukaryotic initiation factor 5A (eIF-5A). The eIF-5A expression is significantly upregulated during the processes of dendritic cells (DCs) maturation and DC-mediated T lymphocyte activation. Hence, a new possibility may be presented by pharmacological interferences with hypusine formation for the modulation of DC function, which causes both T lymphocyte proliferation and activation [94]. Previously, Cheon Kim et al. in their study have reported the differentially expressed Dhps gene, which was observed to be significantly associated with canonical tumorigenesis and tumour’s progression in sporadic colorectal cancers [95].

Therefore, both *Ccr7* and *Dhps* could affect the T lymphocyte proliferation pathway that plays a critical role in the cancer’s progression. Previous studies also indicated that both *Ccr7 *and *Dhps* play an important role in the recruitment of T-cells like diabetogenic Th1 cells, into inflamed islets, and thus in the pathogenesis of type 1 diabetes [96, 97]. So, the above-mentioned studies indeed confirmed the results of this study.

The protein encoded by the laminin subunit gamma 2 (Lamc2) gene is a crucial protein in the basal lamina affecting both the transformed and normal cell’s differentiation, adhesion, migration, survival, and phenotype. The basement membranes play a role as a mechanical barrier to tumour growth. However, these molecules, like laminins, are also considered the important autocrine factors created by various cancers, in order to promote tumorigenesis. The accumulating evidence in this regard revealed that the signalling network mediated by Lamc2 plays key roles in the progression, invasion, and migration of multiple kinds of cancers such as pancreas, stomach, tongue, bladder, and colorectal cancers [98]. Moreover, it is suggested as a potential therapeutic anticancer target to inhibit tumorigenesis [98]. Recently, Kosanam et al. have conducted a comprehensive PDAC tissue proteomic study that demonstrated both the prognostic and diagnostic potentials of Lamc2, and its behaviour in prospective biomarker panels for the improved pancreatic cancer diagnosis [99].

In another study, Jiang et al. have recently reported that there is a significant correlation between *Lamc2* and *Ccr7* of neutrophils in head and neck squamous cell carcinoma after adjusting for tumour purity [100]. The result of this study was in line with ours.

#### The gene regulatory relationship between the genes involved in the statistically significant triplets

The gene regulatory relationship between the genes involved in the statistically significant triplets was traced at this stage. As shown in Fig. 5, the regulatory relationships between *Fhl5 → Peli2*, *Aox1 → Cnksr1*, *Gucy1a3 → Ephx1*, *Svep1 → Peli2*, and *Slc9a9 → Man1a2 *were found with the maximum of seven intermediates. Previous studies have ascertained some of our findings. See below.

Chan et al. in their study demonstrated that Peli2 and Fhl5 genes, as the transcriptional targets of the myocyte enhancer factor 2 (MEF2), are simultaneously up-regulated in human neural progenitor/stem cells (hNPCs) [101]. Previously, it was shown that the MEF2 family of transcription factors is highly expressed in the brain, constituting a key cause of neurodegeneration [102]. In another research, Lee et al. in gene expression dataset GSE29174, have found some breast cancer-related genes after filtering the significantly and differentially expressed genes by fold change. Importantly, those genes, such as *Peli2* and Fhl5, were found to be simultaneously down-regulated in breast cancers [103]. Recently, Namani et al. in their study have discovered the genes related to the *Keap1* mutations, as a result, prognostic genes were observed to be greatly related to the upregulation of the nuclear factor erythroid-2-related factor 2 (NFE2L2/NRF2( path, among the Keap1-mutated Lung adenocarcinoma patients. Surprisingly, using the Position Weight Matrix (PWM) scan and ChIPseek web instruments, it was demonstrated that not only *Peli2* and *Fhl5*, but also *Nqo1* (the corresponding switch gene), as NRF2 binding sites, are involved in this pathway [104].

In this study, two other regulatory relationships were found, which are *Aox1 → Cnksr1 and Svep1 → Peli2*. In a previous study, Namani et al. have found that not only AOX1 serves as an NRF2 binding site, but *Cnksr1*, *Peli2*, and *Svep1 *also are highly correlated with the NRF2 pathway upregulation observed among the Keap1-mutated Lung adenocarcinoma patients [104].

It was demonstrated that *Aox1* serves as a transcriptional target of MEF2 in human neural progenitor/stem cells (hNPCs) [101]. Furthermore, it was shown that a relationship exists between *Aox1* and breast cancer, and a previous study reported that this factor is down-regulated in breast cancer cells [103]. As well, *the Aox1 genewas* reported as an NRF2 binding site in the Keap1-mutated Lung adenocarcinoma patients [104].

Another regulatory relationship was found between *Gucy1a3* and *Ephx1genes*. Accordingly, both *Ephx1* and *Gucy1a3* genes play roles in some biological procedures such as cell cycle/apoptosis androgen signalling, transcriptional regulation, and signal transduction.

The last regulatory relationship was observed between *Slc9a9* and *Man1a2* genes. Namani et al. have also found that not only *Cxcl12* (as a switching gene) serves as an NRF2 binding site, but also both *Slc9a9* and *Man1a2* are highly correlated with the NRF2 pathway upregulation among the KEAP1-mutated Lung adenocarcinoma patients [104].

As indicated in Fig. 5, such gene regulatory relationships are involved in the seven statistically significant triplets. Additionally, such triplets are controlled by *Nqo1*, *Aox1*, *Tspan1*, and *Cxcl12* genes as the switch genes. Interestingly, the central roles of *Nqo1*, *Aox1*, and *Cxcl12* are demonstrated in pancreatic cancer. See below.

Crnogorac-Jurcevic et al. [105] have conducted a large-scale immunoblotting analysis by including more than 900 primary antibodies on pancreatic cancer tissue, chronic pancreatitis, and normal pancreas. Numerous proteins like *Aqx1*, have been shown with different expressions between chronic pancreatitis and pancreatic cancer, indicating their potentially key roles in pancreatic carcinogenesis and as a biomarker for early recognition of pancreatic cancer [106]. The elevated Nqo1 expression in the pancreatic tumour is also represented in numerous early kinds of cancers like pancreatic intraepithelial neoplasia (PanINs) [107]. As mentioned earlier, Nqo1 is overexpressed in pancreatic tumour versus the associated normal tissue; however, catalase expression reduces in comparison to the levels related to normal pancreas tissues. Previously, Shaalan Beg et al. have conducted a clinical trial and as a result, explored a novel Nqo1 bioactivatable drug, named as ARQ761, to target pancreatic cancer treatment and to improve the chemotherapeutic impacts by metabolic modulation in pancreatic cancer [108]. Recently, Garg et al. in their study demonstrated that NFkB activity in pancreatic stellate cells could promote tumour growth by increasing the expression of *Cxcl12*, thereby preventing cytotoxic T-cells from infiltrating the tumour and killing cancer cells. In addition, they suggested some approaches for blocking *Cxcl12* in pancreatic tumour cells, in order to increase antitumor immunity [109].

Although the current study provided new insight into the prognosis of PDAC, more effort should be performed to validate them clinically and extend such findings. We investigated the prognosis of PDAC through suggested switch genes captured in the context of the three-way interaction model. Results were in silico validated through various cancer-related gene expression datasets, and it can be concluded that the suggested switch genes might be significant for PDAC prognosis. Our findings are based on public gene expression datasets (switch genes investigation and validation), so a crucial extension of current work would be to learn whether clinical trials can reiterate the patterns.

## Conclusion and future work

Nowadays, several high-throughput disease-related "omics" datasets are freely available. These datasets comprise valuable information on the disease-related pathways as well as their corresponding gene interactions. In this work, for the first time, we used the three-way interaction model to trace the switch genes that include PC. This approach has some advantages in comparison with the pairwise co-expression analyses; more precisely, the three-way interaction model can deal with the co-expression relationships’ dynamic nature. Therefore, the three-way interaction model can potentially shed light on some cellular alterations causes. In the current research, we suggested several switch genes as diagnostic biomarkers or potential therapeutic anticancer targets for anti-pancreatic cancer therapy. Surprisingly, in previous studies, it has been proved that all of our defined switch genes, including *Lamc2* [99], *Klk1* [77], *Nqo1* [110], *Aox1 *[105], *Tspan1* [111], and *Cxcl12* [112], are closely associated with the pancreatic cancer’s progression. In this regard, some biological processes such as "Response to estradiol", "Embryonic limb morphogenesis pathway", "Positive regulation of cellular amide metabolic process", "The pathway of establishment of the protein localization to plasma membrane", "Type I interferon signalling pathway", "Positive regulation of the developmental growth", and "Regulation of T-cell proliferation" were also indicated to be associated with PDAC. Besides, we have introduced two biologically relevant triplets, namely *Klk1* and {*Gh1*, *Cst3*} and* Lamc2* and {*Dhps*, *Ccr7*}, which may be specifically involved in the onset of pancreatic cancer. Our investigation was performed in the Drug Bank database and showed that some drugs, including Peroxisomal acyl-coenzyme A oxidase 1 and Methocarbamol, are designed for *Aox1* and *Tspan1*, as therapeutic anti pancreatic cancer targets, respectively. The next step may be studying these kinds of drugs on pancreatic cancer cell lines, in order to investigate their exact effect on PDAC suppression.

## Supplementary Information

**Additional file 1.** **S1 Table.** A list of candidate switch genes. **S2 Table.** A list of statistically significant triplets. **S3 Table.** Detailed information of Gene Regulatory Network. **S4 Figure.**The prognostic power of suggested switch genes through glioblastoma, hepatocellular carcinoma and stomach adenocarcinoma as PDAC-unrelated datasets.

## Data Availability

The authors confirm that the data supporting the findings of this study are available within the article and its supplementary materials.
