# Supplementary material for: Novel insight into pancreatic adenocarcinoma pathogenesis using liquid association analysis
Source: BMC Med Genomics. 2022 Feb 18;15:30. doi: 10.1186/s12920-022-01174-3 (PMC8855560; doi:10.1186/s12920-022-01174-3)

S1 Table- A list of candidate switch genes

GenID	logFC	t-test (adj p-value)	dip-test (p-value)
LAMC2	2.90E+00	9.26E-15	1.33E-03
TSPAN1	2.73E+00	9.26E-15	3.52E-03
SERPINB5	2.18E+00	7.33E-12	1.77E-03
FNDC1	1.48E+00	1.28E-06	1.20E-02
COMP	1.47E+00	1.51E-06	2.00E-02
NQO1	1.47E+00	9.39E-11	8.89E-03
CST2	1.23E+00	6.58E-09	8.07E-01
PCDH7	1.01E+00	5.77E-10	4.82E-02
B3GNT3	8.41E-01	2.88E-10	1.71E-02
PRKG1	4.07E-01	5.66E-03	3.74E-02
SLC25A4	-2.27E-01	3.46E-02	4.08E-02
PKI55	-2.64E-01	2.86E-03	4.74E-02
HABP2	-5.26E-01	2.88E-02	1.92E-02
ATP13A4	-5.84E-01	3.59E-03	2.47E-03
CGNL1	-5.88E-01	3.10E-04	3.13E-02
EML5	-7.11E-01	2.19E-07	1.42E-02
RAP1GAP	-7.29E-01	6.53E-05	4.38E-02
SERPINA6	-7.46E-01	2.49E-05	4.26E-02
GRB10	-8.10E-01	3.80E-05	1.87E-03
MUC15	-8.11E-01	3.81E-04	2.25E-02
ABCA5	-9.26E-01	8.60E-07	3.79E-02
SERPINA5	-1.03E+00	5.48E-04	3.38E-03
GPHA2	-1.10E+00	5.47E-05	1.48E-02
ATRNL1	-1.10E+00	4.40E-07	1.82E-02
FAM150B	-1.11E+00	5.15E-07	2.24E-02
ONECUT1	-1.11E+00	8.87E-04	5.59E-03
CXCL12	-1.13E+00	6.37E-10	4.85E-02
GPR98	-1.14E+00	1.12E-07	1.38E-02
SLC39A5	-1.29E+00	1.91E-05	3.26E-03
SYCN	-1.29E+00	2.27E-04	1.49E-02
CTNND2	-1.38E+00	1.21E-06	2.42E-02
KCNJ16	-1.59E+00	6.02E-05	8.96E-04
REG3A	-1.62E+00	9.66E-03	1.31E-02
GATM	-1.66E+00	4.91E-04	2.93E-03
RBPJL	-1.71E+00	1.30E-05	2.56E-02
SLC16A12	-1.87E+00	9.27E-08	3.17E-03
ANPEP	-1.89E+00	3.61E-06	2.17E-02
KLK1	-1.92E+00	4.77E-05	6.05E-04
AOX1	-2.04E+00	1.01E-09	2.81E-02

NR5A2	-2.06E+00	3.32E-06	8.00E-04
KIAA1324	-2.07E+00	4.46E-07	7.15E-03
CELA3B	-2.07E+00	1.82E-04	4.23E-02
EGF	-2.08E+00	3.50E-05	6.51E-03
PDIA2	-2.11E+00	6.92E-06	3.38E-02
REG1P	-2.13E+00	1.34E-03	4.04E-02
CELA2B	-2.28E+00	9.59E-05	7.04E-04
CELA2A	-2.28E+00	1.26E-04	3.75E-02
SERPINI2	-2.48E+00	1.76E-05	1.62E-03
CLPS	-2.54E+00	1.16E-04	4.39E-02
ERP27	-2.59E+00	2.15E-05	1.86E-02
CEL	-2.72E+00	1.05E-04	5.47E-03
PNLIPRP2	-2.82E+00	5.70E-05	2.04E-04
PNLIPRP1	-3.03E+00	2.88E-05	0.00E+00

S2 Table- A list of statistically significant triplets.

num	X1 or X2	X2 or X1	X3	rhodiff	MLA value	wald	p value	bonferroni
1	ADH1B	ACADL	SERPINB5	1.0638	0.3833	37.132	1.10E-09	3.30E-04
2	COLEC11	RGN	LAMC2	1.0635	0.3797	35.4431	2.63E-09	7.89E-04
3	SDC4	SLC2A1	TSPAN1	1.057	0.3829	35.1123	3.11E-09	9.33E-04
4	APPL1	SMARCA2	CXCL12	-1.1878	-0.4255	34.9863	3.32E-09	9.96E-04
5	FAM129A	ADH1B	SERPINB5	1.0886	0.394	34.9372	3.41E-09	1.02E-03
6	RBM4B	PGAP3	ERP27	1.0497	0.3845	34.8683	3.53E-09	1.06E-03
7	GALNT13	LPIN1	CXCL12	1.0829	0.395	34.7898	3.67E-09	1.10E-03
8	ADH1B	PCDHB5	SERPINB5	1.0429	0.3794	34.6667	3.91E-09	1.17E-03
9	PELI2	BOC	CXCL12	-1.125	-0.401	34.6217	4.00E-09	1.20E-03
10	C2orf83	S100A5	EML5	-1.055	-0.3768	34.4867	4.29E-09	1.29E-03
11	LAMB1	ITFG3	LAMC2	1.0634	0.4057	34.2875	4.75E-09	1.43E-03
12	CERS6	SCARNA6	PCDH7	1.1448	0.4074	34.2813	4.77E-09	1.43E-03
13	RPL34	ZNF385D	CXCL12	-1.1062	-0.4016	34.1535	5.09E-09	1.53E-03
14	FAM171B	ATF6	CXCL12	-1.1014	-0.3913	33.3115	7.85E-09	2.36E-03
15	MPP7	KCND3	SERPINB5	1.2776	0.4584	33.2791	7.98E-09	2.39E-03
16	CST3	GH1	KLK1	1.1118	0.4024	33.2717	8.01E-09	2.40E-03
17	LOC692247	TLE2	NQO1	-1.2095	-0.4304	33.1374	8.59E-09	2.58E-03
18	LTBP2	PARP16	LAMC2	1.1021	0.3924	33.1333	8.61E-09	2.58E-03
19	PROX1	RGS13	CXCL12	-1.0554	-0.3791	32.8558	9.93E-09	2.98E-03
20	ADH1A	TPI1	LAMC2	-1.0736	-0.3914	32.8464	9.97E-09	2.99E-03
21	APPL1	SLC9A9	CXCL12	-1.127	-0.4034	32.8202	1.01E-08	3.03E-03
22	NR2C2AP	IER3IP1	KLK1	-1.0983	-0.396	32.667	1.09E-08	3.27E-03
23	APPL1	ZNF385D	CXCL12	-1.205	-0.4359	32.6405	1.11E-08	3.33E-03

24	PRELID2	STX4	CST2	1.1978	0.4248	32.6386	1.11E-08	3.33E-03
25	PROM2	MDK	SERPINB5	1.1664	0.4194	32.6283	1.12E-08	3.36E-03
26	MRPL37	SLFN12	PDIA2	-1.0746	-0.3874	32.5751	1.15E-08	3.45E-03
27	TNFRSF1A	NAV2	NQO1	1.1052	0.3961	32.427	1.24E-08	3.72E-03
28	PELI2	PREX2	LAMC2	1.2116	0.4302	32.3727	1.27E-08	3.81E-03
29	WNK2	PARD6B	SERPINB5	-1.0617	-0.3769	32.0734	1.48E-08	4.44E-03
30	SETD6	SEMA7A	AOX1	-1.0527	-0.3798	32.0259	1.52E-08	4.56E-03
31	PRDM8	TTLL4	NQO1	1.1117	0.4001	32.018	1.53E-08	4.59E-03
32	TMEM56	NAP1L5	CXCL12	-1.0605	-0.3878	32.0158	1.53E-08	4.59E-03
33	FAM178B	ZSCAN21	KLK1	-1.1484	-0.417	31.9522	1.58E-08	4.74E-03
34	CYP3A7	LPIN1	TSPAN1	1.2775	0.4731	31.8831	1.64E-08	4.92E-03
35	CCDC84	MCM3AP	PCDH7	-1.1346	-0.4059	31.6942	1.80E-08	5.40E-03
36	CMA1	C18orf21	MUC15	-1.0726	-0.3836	31.6431	1.85E-08	5.55E-03
37	ANKK1	SPN	ERP27	-1.0996	-0.4033	31.5424	1.95E-08	5.85E-03
38	RGS13	KLF11	CXCL12	-1.0618	-0.3825	31.4821	2.01E-08	6.03E-03
39	ADH1C	APPL1	TSPAN1	1.2281	0.4347	31.4465	2.05E-08	6.15E-03
40	MGLL	CD28	LAMC2	-1.2434	-0.459	31.4109	2.09E-08	6.27E-03
41	ECE1	SUGT1P3	AOX1	1.0805	0.3862	31.3389	2.17E-08	6.51E-03
42	FAM129A	ADH1B	TSPAN1	1.2052	0.4242	31.2929	2.22E-08	6.66E-03
43	ADH1B	APPL1	PCDH7	1.265	0.464	31.1816	2.35E-08	7.05E-03
44	NAP1L5	PHYH	CXCL12	-1.2824	-0.4688	31.1551	2.38E-08	7.14E-03
45	LINC00969	CCNB1	TSPAN1	-1.1422	-0.4133	31.0896	2.46E-08	7.38E-03
46	PPFIA1	CD28	LAMC2	-1.0466	-0.383	31.0627	2.50E-08	7.50E-03
47	SUSD2	PGAP3	KLK1	-1.1593	-0.4162	30.9283	2.68E-08	8.04E-03
48	MPP7	FXYD1	CXCL12	-1.1398	-0.4182	30.9089	2.70E-08	8.10E-03
49	C1orf56	RGS13	CXCL12	-1.1444	-0.4146	30.8774	2.75E-08	8.25E-03
50	RELN	RPL22	LAMC2	1.1398	0.4126	30.863	2.77E-08	8.31E-03
51	CYP2U1	PELI2	AOX1	-1.0723	-0.3786	30.862	2.77E-08	8.31E-03
52	LRIG1	CCDC170	CXCL12	-1.0775	-0.3954	30.8056	2.85E-08	8.55E-03
53	TNFRSF1A	NAV2	CXCL12	-1.1516	-0.41	30.7402	2.95E-08	8.85E-03
54	SEMA4B	OSBPL10	SERPINB5	1.0816	0.3891	30.7269	2.97E-08	8.91E-03
55	ADH1B	GPI	TSPAN1	-1.1496	-0.4048	30.6962	3.02E-08	9.06E-03
56	CTSZ	GMPPA	CXCL12	-1.1399	-0.4079	30.6724	3.05E-08	9.15E-03
57	CNTRL	CLRN2	PDIA2	-1.0979	-0.396	30.6447	3.10E-08	9.30E-03
58	FAM162A	PPCS	ERP27	1.1082	0.3976	30.6365	3.11E-08	9.33E-03
59	ADH1B	MPC1	TSPAN1	1.06	0.3791	30.5745	3.21E-08	9.63E-03
60	CHN2	NLGN1	SERPINB5	1.0859	0.3901	30.5685	3.22E-08	9.66E-03
61	PELI2	FHL5	NQO1	1.0672	0.3849	30.5523	3.25E-08	9.75E-03
62	PITX1	ATXN7	PDIA2	1.0113	0.3772	30.5501	3.25E-08	9.75E-03
63	PCNXL3	MCM7	SERPINB5	1.0352	0.383	30.5049	3.33E-08	9.99E-03
64	FADS6	TBL1Y	CXCL12	-1.118	-0.3974	30.5024	3.33E-08	9.99E-03

65	SLC7A1	DCAF15	CXCL12	-1.0793	-0.3927	30.4094	3.50E-08	1.05E-02
66	LOC100129518	FCGBP	SERPINB5	-1.0693	-0.3822	30.4004	3.51E-08	1.05E-02
67	TMEM208	MRPL24	MUC15	-1.1694	-0.4187	30.2441	3.81E-08	1.14E-02
68	LRIG1	GGTA1P	CXCL12	-1.1199	-0.4058	30.1567	3.99E-08	1.20E-02
69	LOC100131506	RBFOX2	LAMC2	1.2413	0.46	30.1259	4.05E-08	1.22E-02
70	OAS2	NT5C3A	LAMC2	1.0741	0.3816	30.1133	4.08E-08	1.22E-02
71	ITPR3	SETD6	LAMC2	1.1044	0.4098	30.0295	4.26E-08	1.28E-02
72	LRRC42	NCK2	B3GNT3	1.0693	0.3782	29.9558	4.42E-08	1.33E-02
73	ADH1A	APPL1	TSPAN1	1.1745	0.4242	29.9357	4.47E-08	1.34E-02
74	NLGN1	PELI2	TSPAN1	1.0845	0.3806	29.9229	4.50E-08	1.35E-02
75	NLGN1	GNMT	TSPAN1	1.1557	0.4066	29.9218	4.50E-08	1.35E-02
76	TDRD1	SMYD2	CXCL12	1.046	0.3766	29.8766	4.60E-08	1.38E-02
77	LILRB5	PAH	CXCL12	-1.1929	-0.4309	29.8635	4.64E-08	1.39E-02
78	CNKSR1	NSUN6	AOX1	1.1351	0.4016	29.7896	4.82E-08	1.45E-02
79	NSMCE4A	CCL19	CXCL12	-1.0731	-0.3839	29.7786	4.84E-08	1.45E-02
80	PELI2	ZNF385D	CXCL12	-1.2062	-0.437	29.7636	4.88E-08	1.46E-02
81	MPV17L2	ENDOU	MUC15	-1.0681	-0.3849	29.705	5.03E-08	1.51E-02
82	CTSG	RAD17	PCDH7	1.0835	0.3909	29.6549	5.16E-08	1.55E-02
83	PARP16	DPP10	LAMC2	-1.1223	-0.3982	29.6527	5.17E-08	1.55E-02
84	PELI2	SMOC2	LAMC2	1.1619	0.4127	29.6363	5.21E-08	1.56E-02
85	GMPPA	CHST11	CXCL12	-1.1017	-0.3944	29.6097	5.28E-08	1.58E-02
86	AKR7A2P1	GNG11	PDIA2	-1.0551	-0.3826	29.4573	5.72E-08	1.72E-02
87	CCR7	DHPS	LAMC2	1.1064	0.3926	29.4321	5.74E-08	1.72E-02
88	ZNF493	LSAMP	TSPAN1	1.0956	0.3994	29.4489	5.74E-08	1.72E-02
89	APPL1	NXPE3	CXCL12	-1.109	-0.3967	29.4295	5.80E-08	1.74E-02
90	ECE1	GNB1	AOX1	-1.1243	-0.4055	29.4183	5.83E-08	1.75E-02
91	NLGN1	GNMT	NQO1	1.0676	0.3828	29.4101	5.86E-08	1.76E-02
92	KLRG1	SNIP1	AOX1	1.0865	0.3908	29.3866	5.93E-08	1.78E-02
93	RNF145	DHPS	NQO1	1.0961	0.3926	29.3754	5.96E-08	1.79E-02
94	FUNDC2P2	OR2L8	MUC15	-1.1217	-0.4064	29.3591	6.01E-08	1.80E-02
95	PRUNE	ACYP2	EML5	1.2996	0.4528	29.3489	6.05E-08	1.82E-02
96	CTSG	GRSF1	PCDH7	1.1821	0.4245	29.3221	6.13E-08	1.84E-02
97	PELI2	SMARCA2	LAMC2	1.2066	0.424	29.2957	6.21E-08	1.86E-02
98	ADH1C	XPA	TSPAN1	1.204	0.4201	29.2608	6.33E-08	1.90E-02
99	NT5C3A	XAF1	LAMC2	1.0619	0.377	29.2496	6.36E-08	1.91E-02
100	RELN	RNF40	CXCL12	1.0577	0.3793	29.2102	6.49E-08	1.95E-02
101	DAP	STAT1	PDIA2	-1.0711	-0.3849	29.1303	6.77E-08	2.03E-02
102	TMED6	DCAF4	TSPAN1	-1.0629	-0.3792	29.1236	6.79E-08	2.04E-02
103	TMEM56	NHSL2	EML5	-1.0837	-0.391	29.1105	6.84E-08	2.05E-02
104	LGALS2	RIPK4	LAMC2	-1.131	-0.4023	29.0982	6.88E-08	2.06E-02
105	LINC00671	C8orf59	LAMC2	-1.1442	-0.4057	29.0963	6.89E-08	2.07E-02

106	LRIG1	FILIP1	AOX1	-1.1178	-0.4071	29.0933	6.90E-08	2.07E-02
107	EIF4EBP2	ICT1	B3GNT3	-1.1027	-0.4092	29.0885	6.91E-08	2.07E-02
108	OSBPL10	ADH1B	SERPINB5	-1.113	-0.3988	29.0299	7.13E-08	2.14E-02
109	ADH1B	ACADL	TSPAN1	1.1717	0.4139	29.0233	7.15E-08	2.15E-02
110	C6orf201	LRFN5	CXCL12	-1.088	-0.3928	29.0168	7.18E-08	2.15E-02
111	ADH1B	MPC1	PCDH7	1.1118	0.4082	29.0148	7.18E-08	2.15E-02
112	PELI2	FHL5	AOX1	-1.2243	-0.4384	28.9932	7.26E-08	2.18E-02
113	ADH1B	TPI1	LAMC2	-1.1117	-0.4092	28.9916	7.27E-08	2.18E-02
114	HOXA7	FLJ45721	LAMC2	-1.0765	-0.3846	28.9655	7.37E-08	2.21E-02
115	SRA1	GJA10	LAMC2	-1.1447	-0.4154	28.896	7.64E-08	2.29E-02
116	ADH1A	MPC1	PCDH7	1.0869	0.3998	28.8896	7.66E-08	2.30E-02
117	LAMB1	ZDHHC4	CXCL12	-1.2848	-0.4636	28.8799	7.70E-08	2.31E-02
118	FUS	RSL1D1	B3GNT3	1.0346	0.3925	28.8458	7.84E-08	2.35E-02
119	FAAH2	LAX1	CXCL12	-1.1198	-0.3975	28.8387	7.87E-08	2.36E-02
120	TLCD1	LIF	SERPINB5	1.0506	0.3783	28.8238	7.93E-08	2.38E-02
121	ADH1B	PELI2	PCDH7	1.1553	0.4248	28.815	7.96E-08	2.39E-02
122	GLTSCR2	LOC692247	NQO1	-1.1836	-0.425	28.8067	8.00E-08	2.40E-02
123	FXN	MOSPD3	NQO1	-1.2041	-0.4296	28.7935	8.05E-08	2.42E-02
124	AHNAK2	LETM2	CST2	1.0869	0.3937	28.7933	8.05E-08	2.42E-02
125	LRP5	LAP3	ERP27	-1.1007	-0.3928	28.7604	8.19E-08	2.46E-02
126	PSMB9	CERK	TSPAN1	-1.075	-0.3964	28.7223	8.35E-08	2.51E-02
127	IRF6	LGALS2	TSPAN1	-1.1669	-0.4222	28.7135	8.39E-08	2.52E-02
128	TCERG1L	SIGLECP3	B3GNT3	1.1701	0.4203	28.6597	8.63E-08	2.59E-02
129	SNRNP200	SLC9A3R2	CST2	1.2267	0.4341	28.6018	8.89E-08	2.67E-02
130	NAP1L5	ITGB5	CXCL12	1.2299	0.443	28.5988	8.90E-08	2.67E-02
131	LOC100131506	TUBB	LAMC2	1.0714	0.3938	28.573	9.02E-08	2.71E-02
132	CSNK1D	KRT6A	CST2	1.2535	0.4477	28.5227	9.26E-08	2.78E-02
133	CD1C	ABCG1	CXCL12	1.0594	0.3801	28.5097	9.32E-08	2.80E-02
134	PELI2	FHL5	TSPAN1	1.178	0.413	28.4857	9.44E-08	2.83E-02
135	ARFGEF2	PBX1	CXCL12	1.0947	0.3929	28.4823	9.46E-08	2.84E-02
136	LILRB2	FAM209B	PDIA2	1.1357	0.3982	28.4592	9.57E-08	2.87E-02
137	MAN1C1	PELI2	AOX1	-1.0847	-0.3854	28.4498	9.62E-08	2.89E-02
138	EML3	MDH2	AOX1	-1.0399	-0.389	28.4377	9.68E-08	2.90E-02
139	AQP6	RPL13AP20	CXCL12	1.046	0.3774	28.4328	9.70E-08	2.91E-02
140	LINC00114	MUC13	KLK1	-1.0817	-0.3762	28.4316	9.71E-08	2.91E-02
141	LIPM	FAM86C1	EML5	1.0613	0.3822	28.4311	9.71E-08	2.91E-02
142	SUSD2	PGAP3	PDIA2	-1.181	-0.4124	28.43	9.71E-08	2.91E-02
143	VSIG4	AK3	KLK1	-1.0916	-0.3944	28.4227	9.75E-08	2.93E-02
144	NCKAP5	USF1	PDIA2	1.075	0.3874	28.409	9.82E-08	2.95E-02
145	NAP1L5	GKAP1	LAMC2	1.179	0.4218	28.3965	9.88E-08	2.96E-02
146	ZNF593	PITX1	ERP27	1.0687	0.3791	28.3954	9.89E-08	2.97E-02

147	SEC23B	PPIL6	AOX1	1.0843	0.3921	28.3834	9.95E-08	2.99E-02
148	F13A1	LRIG1	LAMC2	1.1271	0.3967	28.3791	9.97E-08	2.99E-02
149	EPHX1	GUCY1A3	TSPAN1	1.1812	0.4154	28.3721	1.00E-07	3.00E-02
150	LRP5	LAP3	KLK1	-1.0518	-0.382	28.3467	1.01E-07	3.03E-02
151	LHFP	MPP7	AOX1	-1.1004	-0.399	28.3318	1.02E-07	3.06E-02
152	SNORD13P2	SCARNA6	PCDH7	1.1038	0.3914	28.3316	1.02E-07	3.06E-02
153	ADH1C	MPP7	TSPAN1	1.1833	0.4223	28.3073	1.04E-07	3.12E-02
154	SLFN5	SRGAP3	PDIA2	1.0777	0.382	28.298	1.04E-07	3.12E-02
155	ADH1A	ACADL	TSPAN1	1.1045	0.4004	28.2955	1.04E-07	3.12E-02
156	KBTBD12	LPIN1	B3GNT3	1.1019	0.3941	28.293	1.04E-07	3.12E-02
157	TDGF1	TMEM133	CXCL12	-1.1835	-0.4265	28.2913	1.04E-07	3.12E-02
158	ZNF596	EML2	LAMC2	-1.0994	-0.3965	28.2867	1.05E-07	3.15E-02
159	MBD6	MTFR1L	SERPINB5	-1.066	-0.3828	28.2379	1.07E-07	3.21E-02
160	SEPT9	NAV2	CXCL12	-1.06	-0.3804	28.2251	1.08E-07	3.24E-02
161	ADH1C	PELI2	TSPAN1	1.1302	0.3955	28.1936	1.10E-07	3.30E-02
162	FAM129A	ZNF831	CXCL12	-1.1497	-0.4138	28.1917	1.10E-07	3.30E-02
163	IRAK2	CH25H	LAMC2	-1.1733	-0.4194	28.186	1.10E-07	3.30E-02
164	EFNA5	PLCXD3	NQO1	-1.0788	-0.3851	28.1852	1.10E-07	3.30E-02
165	ARHGEF3	ARHGAP9	CXCL12	-1.1605	-0.4114	28.1517	1.12E-07	3.36E-02
166	UTRN	DDX18	LAMC2	-1.1291	-0.3981	28.1484	1.12E-07	3.36E-02
167	CHL1	RPL22	CXCL12	-1.0896	-0.3954	28.1476	1.12E-07	3.36E-02
168	MDH2	STAT1	ERP27	-1.0646	-0.3788	28.1412	1.13E-07	3.39E-02
169	FAM84B	CDK1	CXCL12	-1.1483	-0.4124	28.1293	1.13E-07	3.39E-02
170	RPSAP52	ATXN7	MUC15	1.1402	0.4078	28.0777	1.17E-07	3.51E-02
171	NAP1L5	PHYH	LAMC2	1.1823	0.4148	28.0626	1.17E-07	3.51E-02
172	LYPD1	NR3C2	NQO1	-1.0863	-0.3891	28.0475	1.18E-07	3.54E-02
173	MPC1	C7	LAMC2	1.0648	0.3843	28.028	1.20E-07	3.60E-02
174	ERBB2	HSPB2-C11orf52	NQO1	1.1018	0.3934	28.0178	1.20E-07	3.60E-02
175	PELI2	SVEP1	AOX1	-1.2403	-0.44	27.9954	1.22E-07	3.66E-02
176	BRI3	EIF3J	AOX1	1.0257	0.382	27.8907	1.28E-07	3.84E-02
177	NEGR1	PELI2	TSPAN1	1.0931	0.3805	27.8886	1.29E-07	3.87E-02
178	ZNF530	PPFIA1	LAMC2	1.182	0.4246	27.8845	1.29E-07	3.87E-02
179	FAM129A	ADH1A	TSPAN1	1.0844	0.3981	27.882	1.29E-07	3.87E-02
180	C1orf186	MYOM1	CXCL12	-1.0704	-0.3831	27.8703	1.30E-07	3.90E-02
181	GGTA1P	PELI2	CXCL12	-1.0888	-0.3936	27.8466	1.31E-07	3.93E-02
182	MMP19	PPP6C	MUC15	-1.0828	-0.3954	27.8445	1.31E-07	3.93E-02
183	CCR7	IFI27	LAMC2	-1.1071	-0.4012	27.8367	1.32E-07	3.96E-02
184	HACE1	MXD3	CST2	1.1078	0.394	27.8358	1.32E-07	3.96E-02
185	LTN1	NAP1L5	PCDH7	1.0538	0.3781	27.8034	1.34E-07	4.02E-02
186	DCAF4	HID1	LAMC2	-1.1348	-0.3972	27.7852	1.36E-07	4.08E-02

187	HOXD10	EPX	LAMC2	-1.2014	-0.4287	27.7595	1.37E-07	4.11E-02
188	FAM122A	NAP1L5	PCDH7	1.0512	0.3788	27.7508	1.38E-07	4.14E-02
189	PPP1R16B	C2CD4B	EML5	-1.2574	-0.4464	27.7422	1.39E-07	4.17E-02
190	FNDC3A	CD247	EML5	-1.1131	-0.3904	27.6714	1.44E-07	4.32E-02
191	NCKAP5	OAS1	PDIA2	1.1404	0.4102	27.6549	1.45E-07	4.35E-02
192	MSRA	PRPH2	EML5	-1.2214	-0.4326	27.6375	1.46E-07	4.38E-02
193	PSMB9	CERK	LAMC2	-1.1112	-0.3953	27.6335	1.47E-07	4.41E-02
194	OMD	PELI2	CXCL12	-1.0776	-0.379	27.6241	1.47E-07	4.41E-02
195	TPI1	C7	LAMC2	-1.0588	-0.4011	27.6224	1.47E-07	4.41E-02
196	LRIG1	OMD	CXCL12	-1.0545	-0.3919	27.616	1.48E-07	4.44E-02
197	SETBP1	FER1L4	TSPAN1	-1.0589	-0.3803	27.6139	1.48E-07	4.44E-02
198	DPAGT1	CD38	LAMC2	1.1282	0.3908	27.6133	1.48E-07	4.44E-02
199	KRT80	TPI1	LAMC2	1.0583	0.4141	27.6001	1.49E-07	4.47E-02
200	MAN1A2	SLC9A9	CXCL12	-1.1935	-0.4272	27.5945	1.50E-07	4.50E-02
201	EXOSC6	TFCP2L1	CST2	-1.1951	-0.4413	27.5882	1.50E-07	4.50E-02
202	PKP3	BTG2	NQO1	-1.1958	-0.4295	27.5804	1.51E-07	4.53E-02
203	LOC692247	TLE2	SERPINB5	-1.2036	-0.4261	27.5661	1.52E-07	4.56E-02
204	NLGN1	PELI2	SERPINB5	1.1508	0.413	27.5293	1.55E-07	4.65E-02
205	LOC100131506	FRMD6	LAMC2	1.1004	0.4038	27.5247	1.55E-07	4.65E-02
206	PELI2	PREX2	TSPAN1	1.0849	0.3798	27.5145	1.56E-07	4.68E-02
207	LPIN1	ARHGEF38	TSPAN1	1.1555	0.4222	27.5041	1.57E-07	4.71E-02
208	TMEM176A	ZNF85	CXCL12	-0.9847	-0.3812	27.5017	1.57E-07	4.71E-02
209	DPH2	CHRD	TSPAN1	-1.2318	-0.421	27.4925	1.58E-07	4.74E-02
210	TROAP	TDG	AOX1	-1.0634	-0.3831	27.4881	1.58E-07	4.74E-02
211	CTSG	C17orf75	PCDH7	1.1704	0.4234	27.47	1.60E-07	4.80E-02
212	CYP3A7	LPIN1	CST2	1.0314	0.3783	27.4646	1.60E-07	4.80E-02
213	LINC00346	CD6	CXCL12	1.0925	0.3873	27.4496	1.61E-07	4.83E-02
214	CYB561A3	CDKN2AIPNL	PDIA2	-1.1468	-0.4129	27.4339	1.63E-07	4.89E-02
215	PCDHB5	LOC692247	SERPINB5	-1.1433	-0.4056	27.4337	1.63E-07	4.89E-02
216	ZFP3	SPAG17	PCDH7	-1.1935	-0.4279	27.4313	1.63E-07	4.89E-02
217	chr17:76886720-76897910	KLK6	PCDH7	1.0451	0.3763	27.4302	1.63E-07	4.89E-02
218	CMA1	C18orf21	KLK1	-1.1229	-0.3964	27.4078	1.65E-07	4.95E-02
219	PRDM5	ADH1C	TSPAN1	1.1848	0.42	27.406	1.65E-07	4.95E-02
220	IRF9	LRFN5	B3GNT3	-1.0665	-0.3974	27.4003	1.65E-07	4.95E-02
221	SEPHS1	SFT2D2	CST2	-1.1002	-0.4052	27.3932	1.66E-07	4.98E-02

S3 Table

Detailed information of Gene Regulatory Network

S node	T node	MI
AOX1	PRKCE	0.44
PRKCE	HERC1	0.38
HERC1	MYCBP2	0.6
MYCBP2	CNKSR1	0.5
FHL5	ACADL	0.41
ACADL	MIR217	0.61
MIR217	PELI2	0.61
GUCY1A3	DSEL	0.38
DSEL	FAR2	0.39
FAR2	BACE1	0.61
BACE1	EPHX1	0.63
SVEP1	FHL5	0.56
FHL5	ACADL	0.41
ACADL	MIR217	0.61
MIR217	PELI2	0.61
SLC9A9	ITPR1	0.68
ITPR1	PI4KA	0.51
PI4KA	INPP4A	0.5
INPP4A	CSK	0.48
CSK	ADRBK1	0.55
ADRBK1	B3GALNT2	0.6
B3GALNT2	MAN1A2	0.62

S4 Figure

The prognostic power of suggested switch genes through glioblastoma, hepatocellular carcinoma and stomach adenocarcinoma as PDAC-unrelated datasets.

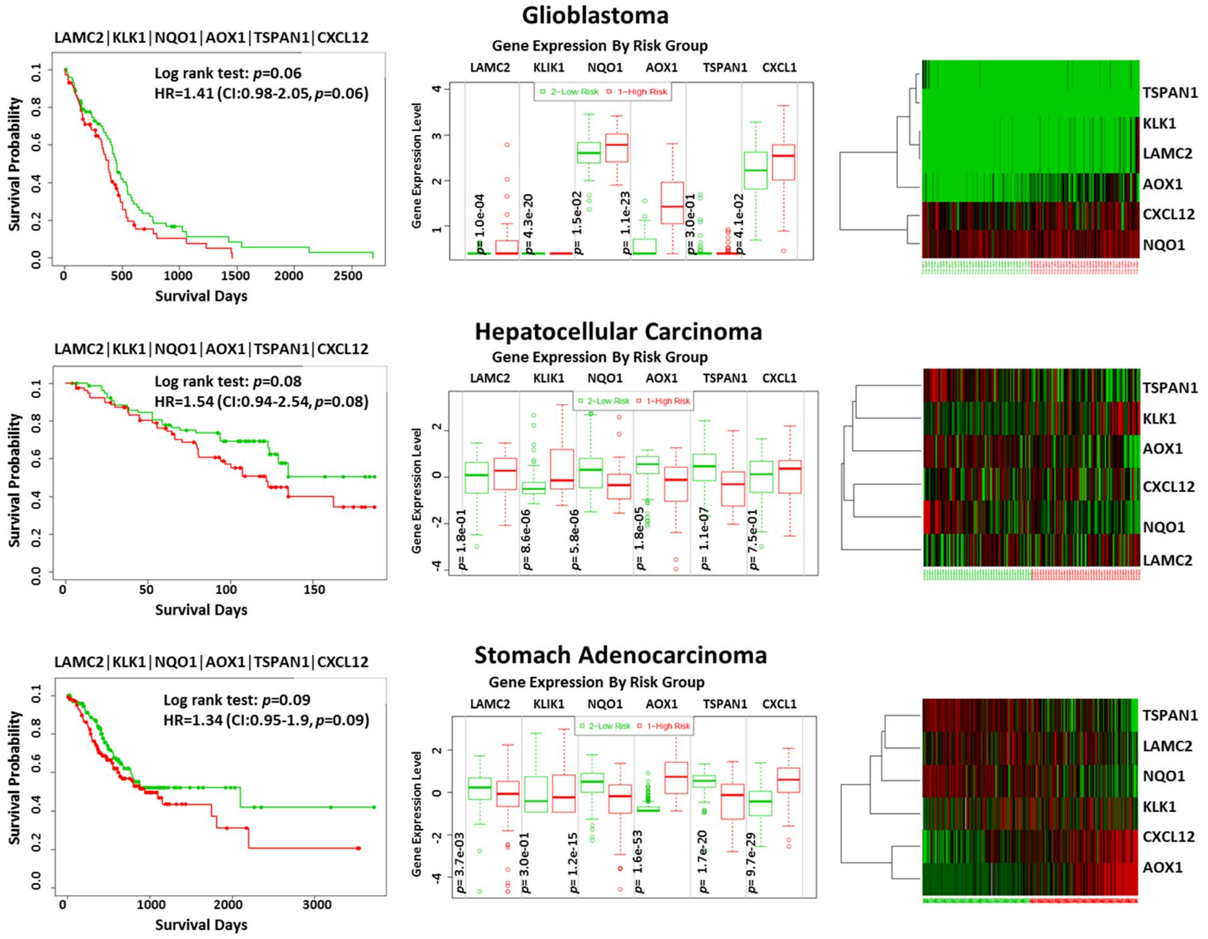

Supplement: Supplementary file 1 — Additional file 1. S1 Table. A list of candidate switch genes. S2 Table. A list of statistically significant triplets. S3 Table. Detailed information of Gene Regulatory Network. S4 Figure.The prognostic power of suggested switch genes through glioblastoma, hepatocellular carcinoma and stomach adenocarcinoma as PDAC-unrelated datasets. [file 12920_2022_1174_MOESM1_ESM.pdf]
